# MET inhibitor, capmatinib overcomes osimertinib resistance via suppression of MET/Akt/snail signaling in non-small cell lung cancer and decreased generation of cancer-associated fibroblasts

**DOI:** 10.18632/aging.202547

**Published:** 2021-02-17

**Authors:** Kaibin Zhu, Zhonghua Lv, Jinsheng Xiong, Hongshan Zheng, Sibin Zhang, Hua Jin, Lei Yu, Zhenzhe Li, Jixing Zhang, Chenlong Li, Peng Liang

**Affiliations:** 1 Department of Thoracic Surgery, Harbin Medical University Cancer Hospital, Harbin 150001, Heilongjiang, China; 2 Department of Neurosurgery, Harbin Medical University Cancer Hospital, Harbin 150001, Heilongjiang, China

**Keywords:** osimertinib resistance, non-small cell lung cancer (NSCLC), cancer-associated fibroblasts (CAFs), MET associated signaling, capmatinib

## Abstract

Background: Patients with non-small cell lung cancer (NSCLC) initially responding to tyrosine kinase inhibitors (TKIs) eventually develop resistance due to accumulating mutations in the EGFR and additional lesser investigated mechanisms such as the participation of the tumor microenvironment (TME).

Methods: Here, we examined the potential for MET inhibitor capmatinib for the treatment of osimertinib-resistant NSCLCs and normalizing the TME.

Results: We first established that HCC827 and H1975 cells showed increased resistance against osimertinib when co-cultured with CAFs isolated from osimertinib-resistant patients. Additionally, we showed that CAFs promoted epithelial-mesenchymal transition (EMT) and self-renewal ability in both HCC827 and H1975 cells. We subsequently found that both CAF-cultured HCC827 and H1975 showed a significantly higher expression of MET, Akt, Snail and IL-1β, which were associated with survival and inflammatory responses. These cells in turn, promoted the generation of CAFs from normal lung fibroblasts. Subsequently, we observed that the treatment of capmatinib resulted in the re-sensitization of CAF-co-cultured H1975 and HCC827 to osimertinib, in association with reduced EMT and self-renewal ability. MET-silencing experiment using siRNA supported the observations made with capmatinib while with a greater magnitude. MET-silenced cell exhibited a severely hindered expression of inflammatory markers, IL-1β and NF-κB; EMT markers, Snail and Vimentin, while increased E-cadherin. Finally, we demonstrated that the combination of capmatinib and osimertinib led to an increased tumor inhibition and significantly lower number of CAFs within the patient derived xenograft (PDX) model.

Conclusion: Taken together, our findings suggested that an increased MET/Akt/Snail signaling was induced between the NSCLC cells and their TME (CAFs), resulting in osimertinib resistance. Suppression of this pathway by capmatinib may bypass the EGFR activating mutation and overcomes osimertinib resistance by targeting both tumor cells and CAFs.

## INTRODUCTION

Activating EGFR mutation represents one of the key driver oncogenes in patients with non-small cell lung cancer. The discovery of gefitinib, the first generation of EGFR-tyrosine kinase inhibitors (TKIs) paved the way for targeted therapy for NSCLC patients with activating EGFR mutations (L858R and exon-19 deletion). Unfortunately, most of the initial TKI responders often experience disease relapse due to the acquisition of additional mutations [1]. The acquisition of EGFR T790M mutation was identified to be the cause for resistance against the gefitinib found in approximately 50% of all initial responders [2, 3]. Subsequently, the third generation TKIs, AZD9291 (osimertinib) which irreversibly binds to and inactivate EGFR T790M was developed and provided impressive responses in EGFR T790M-positive patients [4, 5]. Unfortunately, patients who received osimertinib eventually develop resistance via acquired additional EGFR C797S mutation [6, 7]. More importantly, additional activation of other survival signaling pathways such as MET and associated signaling, were also observed to be activated in patients who picked up the C797S mutation [8–10]. Thus, TKI treatment may inevitably force the tumor cells to adapt and acquire resistance for survival. The evaluation of multi-targeted approaches should therefore be considered.

One of the established facilitators of lung cancer progression with the tumor microenvironment (TME) is the cancer-associated fibroblasts (CAFs). CAFs are originally normal residential (or recruited) fibroblasts which are educated and transformed by the lung cancer cells to have the abilities to promote tumor progression [11]. Within the TME, CAFs secrete inflammatory cytokines which enhance survival signaling pathways and resist drug treatments. For example, HGF (hepatocyte growth factor) produced by CAFs activates and enhances PI3K/ATK signaling in the tumor cells, promoting cancer cell survival and resistance against treatments [12, 13]. Also, pro-inflammatory cytokines such as IL-6 and IL8 are released by CAFs to enhance chemoresistance by activating STAT3 signaling (another powerful pro-survival pathway) in lung cancer cells [11, 14]. Therefore, this two-way cellular communication within the TME significantly favors cancer progression. Targeting lung cancer cells specifically is becoming evidently inadequate, as reflected by the acquired TKI resistance through different venues. The interruption of the communications between lung cancer cells and CAFs should be considered when developing therapeutic interventions.

Here, we first demonstrated that co-culturing with CAFs from osimertinib-resistant patients, NSCLC cell lines, H1975 and HCC827 became more osimertinib resistant, and showed enhanced ability to form colonies, tumor spheres and migrate. CAF-mediated effects were accompanied with increased expression in EMT, stemness and survival pathways including, EGFR, MET, Akt and β-catenin. In parallel, osimertinib-resistant NSCLC cells promoted the generation of CAFs via secreting CAF transforming cytokines, PDGF and TGFβ, favoring disease progression. We demonstrated that disrupting this tumor-CAF communication loop by capmatinib and osimertinib combination regimen (suppressing MET/Akt/EGFR) overcame osimertinib resistance. Finally, we validated *in vitro* data using a patient-derived xenograft mouse model and showed the efficacy of capmatinib combined with osimertinib suppressed tumor growth of osimertinib-resistant cancer cells. Our observations provided preclinical evidence for treating patients with osimertinib resistant NSCLC via dual inhibition of EGFR/MET and reducing the generation of CAFs.

## RESULTS

### Cancer associated fibroblasts (CAFs) promote malignant characteristics in non-small cell lung cancer cell lines

**Figure 1:**
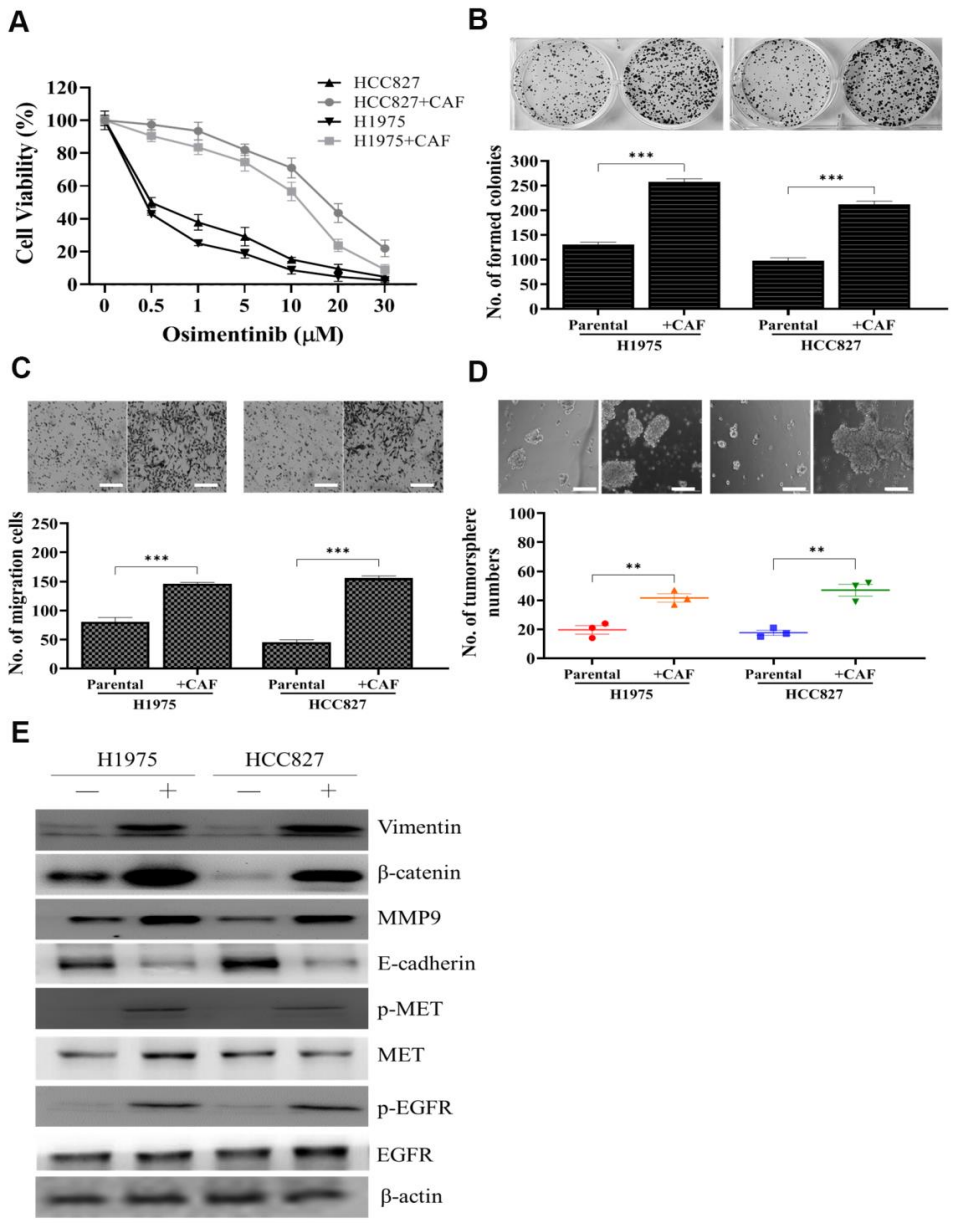
**Osimertinib resistant clinical samples of CAF increased oncogenic properties in NSCLC cells.** (**A**) Cell viability assay showed that CAF-coculture led to an increased osimertinib in both H1975 and HCC827 cells as compared to their parental counterparts. In the presence of CAFs, both H1975 and HCC827 cells exhibited a significantly enhanced ability to form colonies (**B**) migrate (**C**) and generate tumor spheres (**D**). (**E**) Western blots analysis of CAF-cocultured H1975 and HCC827 cells indicated a prominently increased activity of EGFR, MET, elevated expression of EMT markers, vimentin and MMP9, stemness marker, β-catenin.

We first co-cultured H1975 and HCC827 NSCLC cell lines with clinically isolated CAFs. We found that the presence of CAFs increased osimertinib resistance in both cell lines. For instance, the IC_50_ value for parental HCC827 was estimated 0.5μM but was increased to approximately 12.5μM after co-cultured with CAFs (Figure 1A). In addition, CAF co-culture resulted in the significantly increased abilities to form colonies (Figure 1B), to migrate (Figure 1C) and generate tumor spheres (Figure 1D) in both cell lines. Western blots were used compared parental (-) and CAF (+) co-cultured NSCLC cells. CAF coculture led to an increased activity of EGFR and MET (increased phosphorylated forms of EGFR and MET), increased expression of EMT markers, vimentin, MMP9, while decreased E-cadherin and stemness indicator, β-catenin (Figure 1E).

### Osimertinb resistant NSCLC cells exhibit enhanced MET/Akt promotes the generation of CAFs

**Figure 2:**
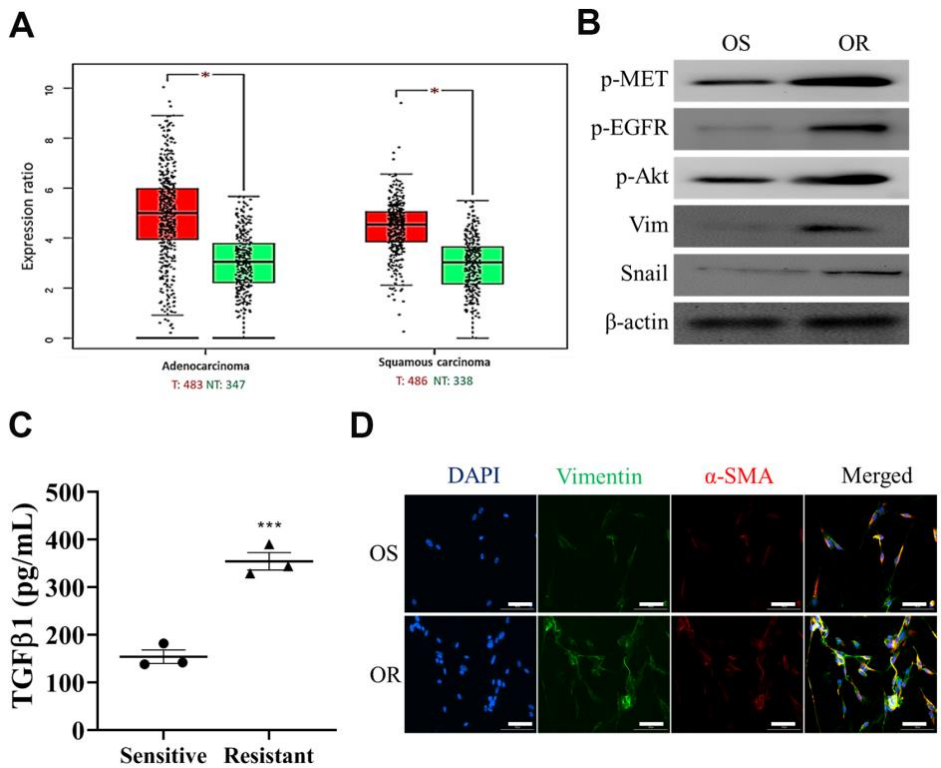
**Osimertinib-resistant cells promoted CAF transformation.** (**A**) Expression profile analysis of TCGA lung cancer cohorts showed that an elevated MET mRNA level in both lung adenocarcinoma and squamous cell lung cancer tissues as compared to the normal lung tissue. *P<0.05. (**B**) Western blots analysis showed that osimertinib resistant (OR) NSCLC samples contained a higher EGFR, Akt and MET activities (p-, phosphorylated form) and higher expression of EMT markers, vimentin and snail, than osimertinib sensitive (OS) counterparts. (**C**) ELISA assay of medium collected from OR and OS cells showed that OR cells produced a significantly higher amount of TGFβ1 as compared to OS cells. (**D**) Immunofluorescence examination of fibroblasts after co-cultured with OR or OS cells. Fibroblasts co-cultured with OR cells showed a markedly stronger fluorescence intensity of both CAF makers, α-SMA (green) and vimentin (red), comparing to fibroblasts co-cultured with OS cells (magnification 200X).

Next, we first demonstrated that an increased expression of MET in both lung adenocarcinoma and lung squamous cell carcinoma (Figure 2A) from TCGA database analysis (TCGA Research Network: https://www.cancer.gov/tcga), indicating that MET activation in both subtypes of lung cancer. We then collected clinical samples of osimertinib sensitive (OS) and resistant (OR) and showed that OR samples contained an enhanced EGFR, MET and Akt activities, evident by the increased phosphorylation of these proteins (Figure 2B); also an increased expression of vimentin and snail was observed in the OR samples as compared to the OS samples (Figure 2B). Furthermore, we demonstrated that OR cells released a significantly higher amount of CAF-transforming cytokine, TGFβ1 than the OS counterparts (Figure 2C). Consistently, OR cells comparatively were more capable of transforming normal lung fibroblasts (NLFs) to CAFs than the OS cells, as reflected by the increased expression of FAP, vimentin and α-SMA in the OR co-cultured CAFs (Figure 2D).

### Capmatinib treatment reduced osimertinib resistance in NSCLC cells via downregulation of MET/Akt/snail expression

**Figure 3:**
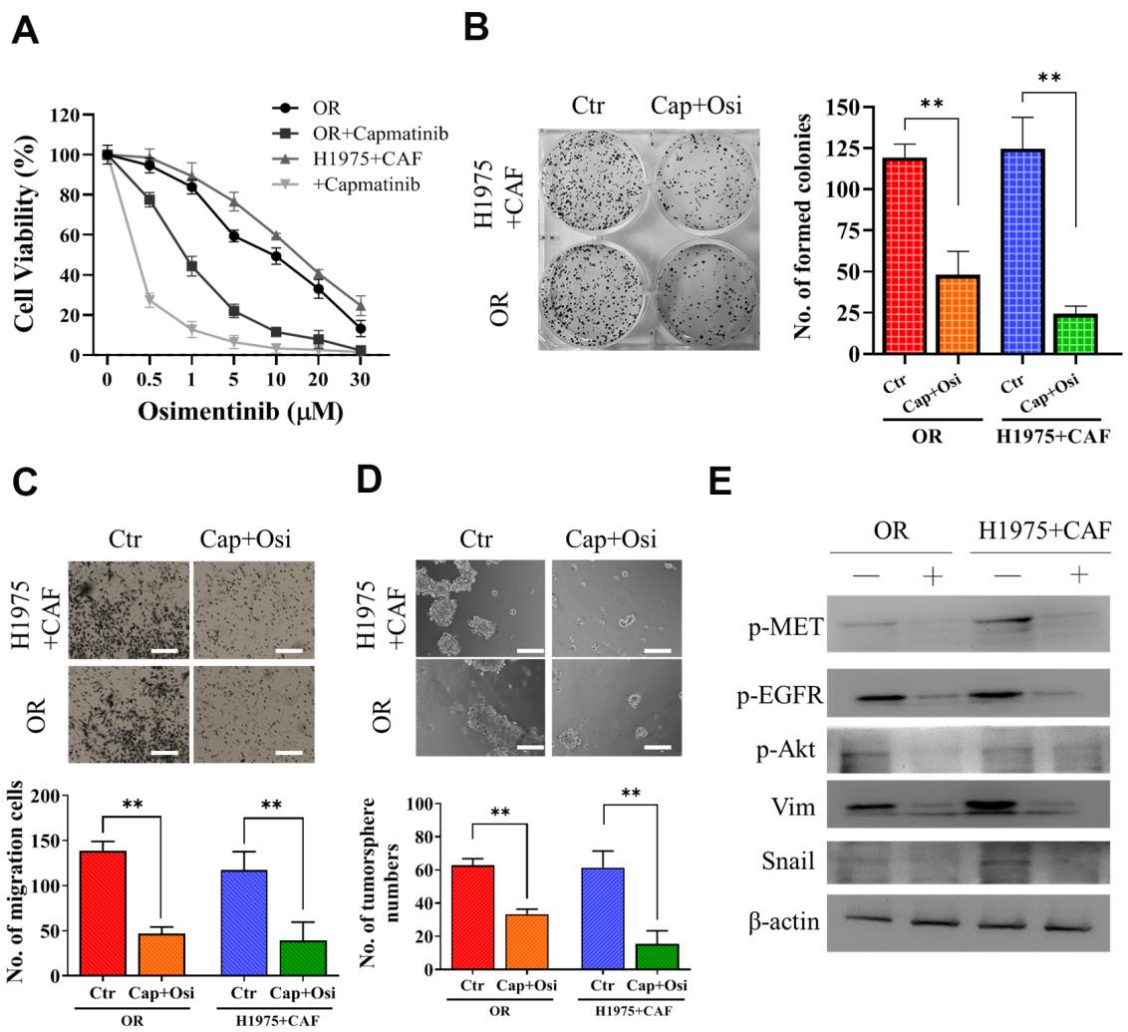
**Capmatinib treatment overcomes osimertinib resistance.** (**A**) Cell viability assay showed that capmatinib treatment (0.5μM, 24h) followed by osimertinib promoted osimertinib sensitivity in both H1975+CAF and OR cells. The combined treatment regimen: capmatinib (0.5μM, 24h) followed by osimertinib (0.25μM, 48h for H1975+CAF and 0.5μM 48h for HCC827+CAF, respectively, significantly reduced the ability of colony formation (**B**) migration (**C**) and tumor sphere generation (**D**). (**E**) Western blot analysis revealed that the sequential capmatinib and osimertinib treated H1975+CAF and OR cells showed a markedly reduced phosphorylation of MET, Akt, EGFR and expression level of vimentin (Vim) and Snail than the untreated counterparts. Lane +, treatment group; lane -, untreated group. **P<0.01; ***P<0.001.

Subsequently, we examined the effect of MET inhibitor, capmatnib, on osimertinib resistance. We found that the addition of capmatinib (0.5μM, 24h) followed by osimertinib could markedly reduced the resistance in both OR and H1975+CAF cells (co-cultured with CAFs) (Figure 3A). Other malignant properties including colony formation (Figure 3B), migration (Figure 3C) and tumor sphere generation (Figure 3D) were also significantly reduced by the sequential treatment of capmatinib (0.5μM, 24h) and osimertinib (0.25μM, 48h for H1975+CAF and 0.5μM 48h for HCC827+CAF, respectively). The western blots of these cell lysates revealed that the activity of MET, EGFR and Akt were significantly reduced along with snail and vimentin (Figure 3E).

### Silencing MET significantly reduced osimertinib resistance

**Figure 4:**
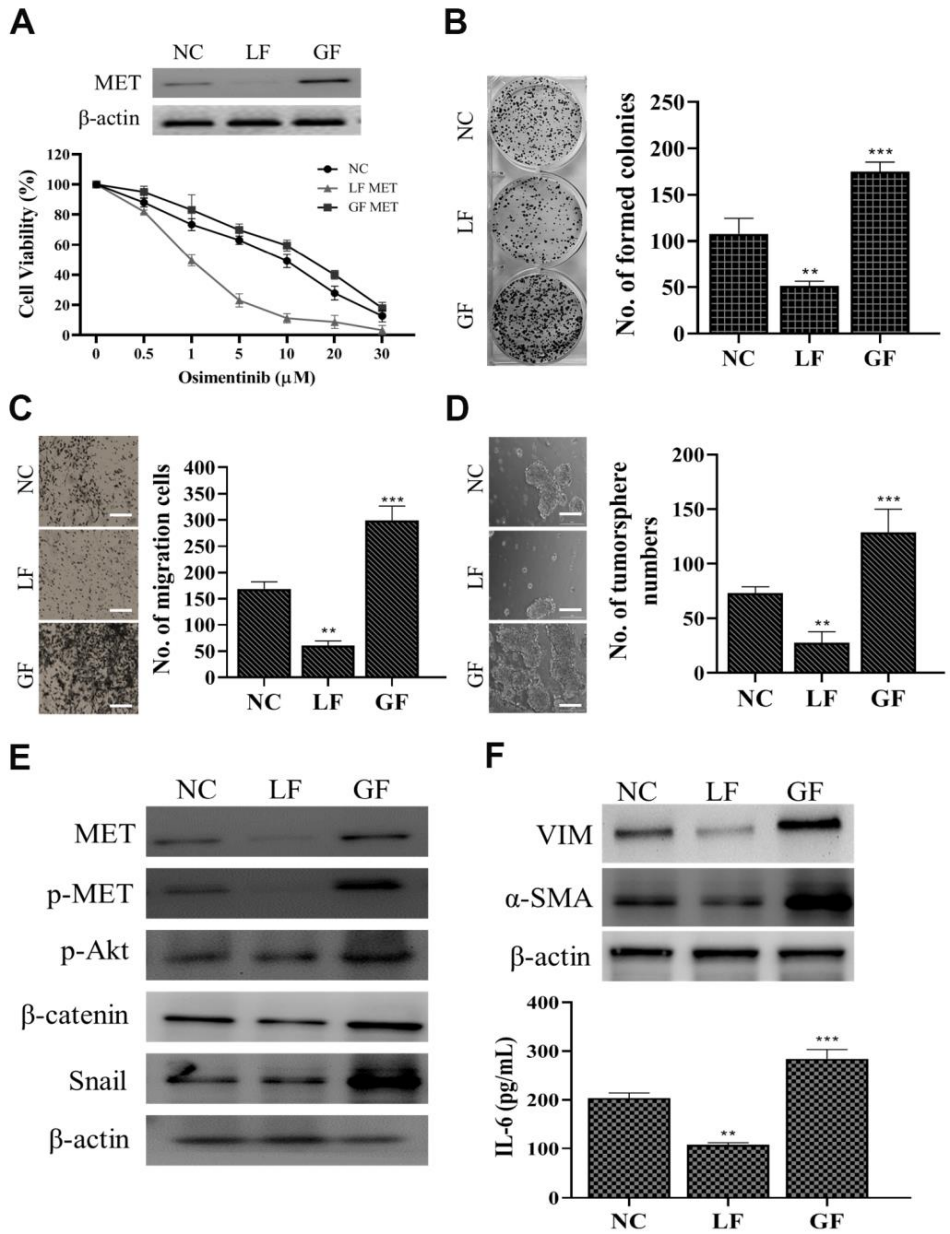
**MET expression is positively correlated with osimertinib resistance in NSCLC cells.** (**A**) MET silencing (loss of function, LF) reduced osimertinib resistance in clinical NSCLC cells (osimertinib resistant cells, OR) and overexpression of MET (gain of function, GF) increased osimertinib resistance. The insert shows the MET expression in LF and GF in the OR cells. NC, normal control (vector control). LF MET reduced colony formation (**B**) migration (**C**) and tumor sphere generation (**D**) in OR cells. (**E**) Comparative western blots of MET LF and GF OR cells. MET-silenced OR cells revealed a clear reduction in the expression of MET, phosphorylated forms of MET and Akt (p-MET and p-Akt respectively), β-catenin, and Snail while the opposite was observed in the MET overexpressing (GF) counterparts. (**F**) CAF co-cultured with MET LF OR cells secreted a significantly lower IL-6 into the culture medium as compared to MET GF OR cells. **P<0.01, ***P<0.001.

After demonstrating capmatinib treatment was able to overcome osimertinib resistance we silenced MET and observed its effects on OR cells. We found that silencing MET (LF= loss of function) led to a prominently increased sensitivity towards osimertinib while restoring MET (GF= gain of function) showed the opposite phenomenon (Figure 4A). In addition, MET-silenced OR cells showed a significantly reduced ability to form colonies (Figure 4B), migrate (Figure 4C) and generate tumor spheres (Figure 4D). Western blots analysis indicated that LF-MET OR cells showed a clear reduction of MET expression and its downstream signaling including p-STAT3, p-Akt, Snail, and β-catenin (Figure 4E). More importantly, MET-silenced OR cells exhibited lost a significantly degree of CAF-transforming capacity as compared to their parental counterparts (Figure 4F), as the expression of VIM and a-SMA were significantly reduced in the fibroblasts post co-culture with MET-silenced OR cells (upper panel, Figure 4F). In addition, the amount of IL-6 released by CAFs co-cultured with GF-MET OR cells was significantly higher as compared to the CAFs co-cultured with LF-MET OR cells (lower panel, Figure 4F).

### The combination of capmatinib and osimertinib overcame osimertinib resistance *in vivo*

**Figure 5:**
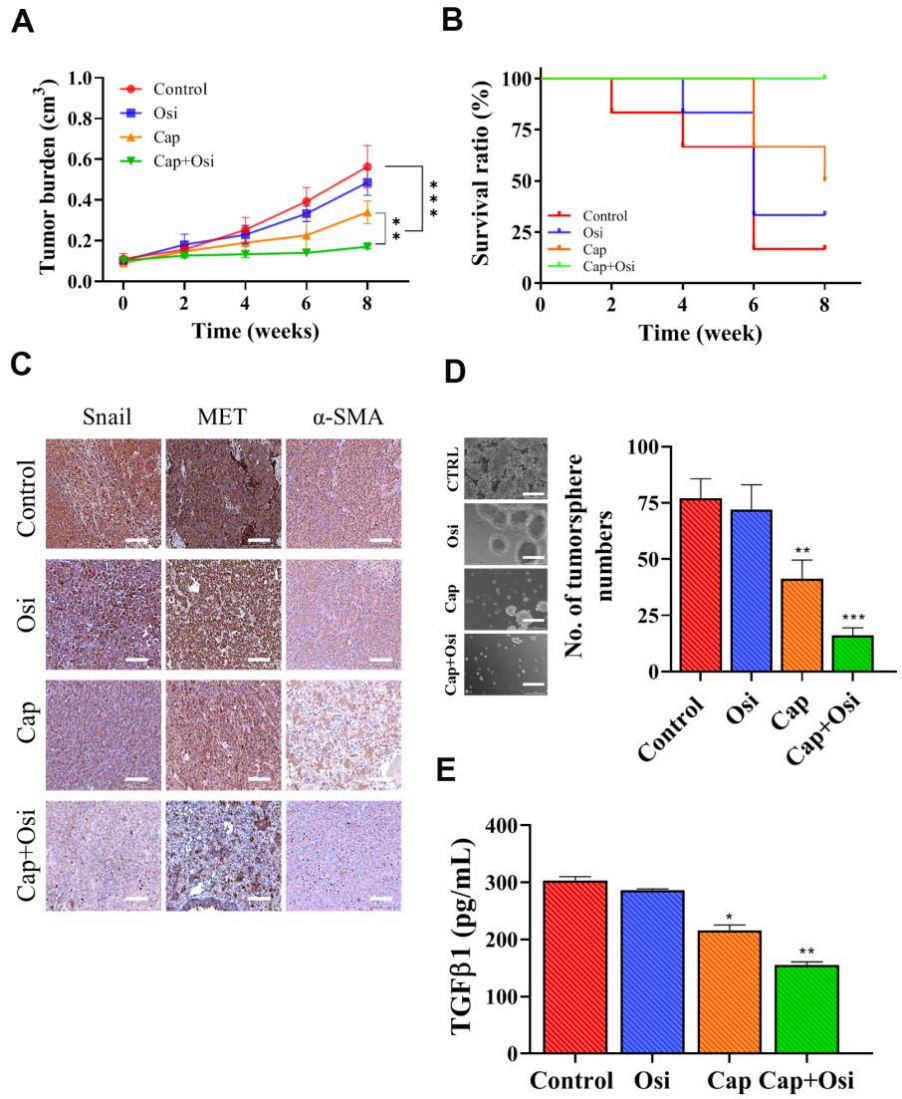
**Combination treatment of capmatinib and osimertinib overcame osimertinib resistance and improved survival in PDX mouse model.** (**A**) Tumor burden over time curve shows that the combination treatment (Cap+Osi) group with the lowest tumor burden followed by capmatinib only (cap) group while no difference between the control and osimertinib (osi) only groups. (**B**) Survival ratio versus time curve indicates that the best survival rate in the following order, combination treatment (cap+osi), capmatinib (cap) only, and lowest in both control and osimertinib (osi) only groups. (**C**) Immunohistochemical analysis of tumor samples. The lowest staining intensity of Snail MET and α-SMA was found in the combination group followed by the capmatinib only samples while the immunostaining of all three markers were highest in the control and osimertinib only tumor sections. (**D**) Comparative tumor formation assay shows that the lowest number of tumor spheres were formed in the samples which received the combination treatment followed by capmatinib only. Tumor samples from control and osimertinib only groups showed similar tumor forming ability. (**E**) ELISA assay of secreted TGFβ1 secretion by the tumor samples with different treatment regimens. The lowest amount of TGFβ1 secreted into the medium was observed in the tumor samples received combination treatment. **P<0.01, ***P<0.001, NS, no significance.

We subsequently validated *in vitro* observations which capmatinib and osimertinib combination re-sensitize NSCLC towards osimertinib treatment, using patient-derived xenograft mouse model. Tumor masses isolated from patients with osimertinib-resistance were subcutaneously injected into NSG mice. We found that the tumor burden in mice which received the combination treatment was statistically lowest when compared with capmatinib alone counterparts while osimertinib alone and vehicle control exhibited similar tumor burden (Figure 5A). In addition, mice treated the combination of capmatinib and osimertinib showed the best survival rate among all groups (Figure 5B). We also harvested the tumor samples and performed immunohistochemical analysis. Indeed, tumor sections from the combination treatment showed the most reduction in the expression of Snail, MET and α-SMA as compared to other sections (Figure 5C). Notably, the expression of these markers between the control and osimertinib single treatment did not differ much, while capmatinib treatment slightly reduced the expression of Snail, MET and α-SMA (Figure 5C). Furthermore, we also compared the tumor sphere forming ability among these tumor samples, In agreement, tumor samples from the combination treatment group showed the lowest ability to form tumor spheres under serum-derived conditions followed by capmatinib only group while no difference between the control and osimertinib alone groups (Figure 5D). In the culture media, we observed the secreted CAF-promoting cytokine, TGFβ1 was the lowest in the samples receiving the combination treatment, followed by the capmatinib only samples while no significant difference between the control and osimertinib only groups (Figure 5E).

## DISCUSSION

The recently developed osimertinib, the third generation of EGFR targeted inhibitor, has shown survival benefits in patients with acquired resistance against both gefitinib and afatinib [15]. Unfortunately, like their predecessors, patients received osimertinib gradually develop resistance via acquiring additional mutant, C797S [16]. The underlying reason for osimertinib appeared to be unclear but in part, we demonstrated that CAFs may contribute to this phenomenon. It is important to find ways to overcome osimertinib resistance in order to help improve the clinical outcomeof these patients. Since the tumor microenvironment has been well established to play a significant role in tumor progression, as well as drug resistance [17, 18], we decided to examine the role of cancer-associated fibroblasts in the development of osimertinib resistance, We found that the CAFs isolated from osimertinib-resistant patients were able to promote osimertinib resistance when co-cultured with H1975 and HCC827 NSCLC cells. The presence of CAF appeared to induce the expression of several oncogenic pathways, such as MET, Akt and EMT/stemness markers, Snail and β-catenin. Increased MET and Akt activity has been associated with acquired resistance against EGFR TKI inhibitors, independent of additional mutations in the EGFR [19, 20]. In addition, our findings suggested that EMT was also enhanced due to the co-culture of CAFs and increased Snail and β-catenin may contribute to the survival advantage of these NSCLC cells [21–23]. In agreement, CAF co-cultured H1975 and HCC827 cells were able to form a significantly higher number of tumor spheres, with the enhanced self-renewal and drug resistant abilities. Our findings strongly suggested that the tumor microenvironmental factor such as CAFs also contributed to osimertinib resistance in NSCLC.

In reverse, we also found that osimertinib-resistant lung cancer cells obtained from the patients enhanced CAF transformation by releasing a significantly higher amount of TGFβ1as compared to the osimertinib-sensitive counterparts. This phenomenon provided confirmation to our notion that the cellular communications between the tumor cells and CAFs played at least in part to promote osimertinib resistance. The increased CAF-transforming ability of these clinical osimertinib NSCLC cells was associated with increased signaling in MET, Akt, EGFR, and vimentin, all of which contributed towards ceullar survival, proliferation and EMT [24, 25]. Based on these findings, we explored the possibility of inhibiting MET signaling using capmatinib in combination with osimertinib. We found that capmatinib treatment followed by osimertinib significantly re-sensitized CAF-trained osimertinib resistant H1975 cells and OR cells (osimertinib-resistant NSCLC obtained from patients). This capmatinib-osimertinib sequential treatment regimen appeared to overcome osimertinib resistance by two major routes. First, the inhibition of multiple oncogenic signaling networks including EGFR, MET, Akt and EMT/stemness signaling, Snail and β-catenin. Second, an indirect way through normalizing the tumor microenvironment, capmatinib-osimertinib combination regimen resulted in the reduced TGFβ1 secretion, which promotes CAF transformation, by the osimertinib-resistant NSCLC.

**Figure 6:**
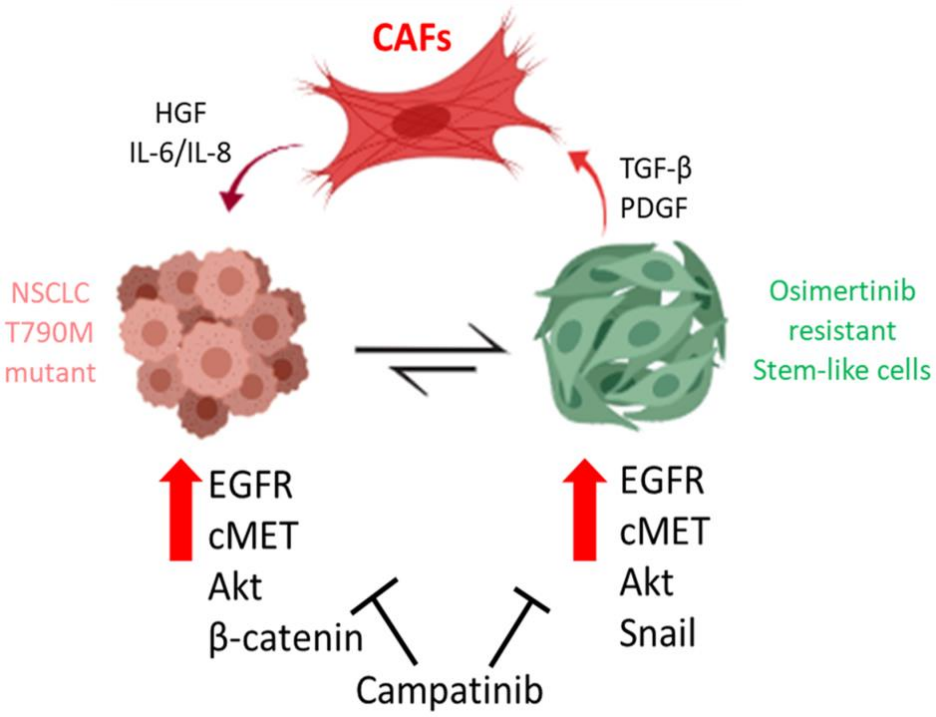
**Cancer associated fibroblasts (CAFs) from osimertinib-resistant patients promoted osimertinib resistance via HGF/MET signaling and induction of epithelial-to-mesenchymal transition (EMT) and transformed NSCLC cells into TKI resistant stem-like cells.** The treatment of capmatinib (MET inhibitor) inhibited CAF-mediated MET/Akt activation bypassing EGFR signaling pathway and re-sensitizing NSCLC towards osimertinib.

Subsequently, we also demonstrated the important function of MET in terms of promoting osimertinib using gain- and loss-of function experiments. Notably, MET silencing led to an increased sensitivity towards osimertinib in the osimertinib resistant (OR) cells. Interestingly, MET expression was previously found to be associated with resistance against gefitinib (the first generation TKI) via activating ERBB3 [26]. According to our experimental findings, we also observed the downregulation of other markers such as Akt and β-catenin. In agreement, a recent study just demonstrated that dual inhibition of MET and PI3K (upstream of Akt) by a small molecule, could suppress NSCLC tumorigenesis [24]. In addition, the suppression of β-catenin expression resulted in the significantly reduced self-renewal ability in the osimertinib-resistant OR cells, making them more sensitive towards the subsequent osimertinib treatment. This finding was partially supported by previous reports where the suppression of β-catenin resulted in the suppression of other signaling networks such as TRAIL, Akt and CDK6 [23, 27]. More importantly, the reduction in Snail expression post MET silencing, could also contribute towards osimertinib re-sensitization in OR cells. EMT markers such as Snail and Twist1 have been implicated in the acquisition of chemotherapeutic resistance [28–30]. These *in vitro* observations were confirmed in our PDX mouse model where the combination treatment of capmatinib and osimertinib provided the most prominent tumor suppressive effects among osimertinib only and capmatinib only regimens. In addition, mice which received the combination treatment showed the best survival rate as compared to all the other groups. Collectively, this study provided preclinical support for using capmatinib for the treatment of osimertinib-resistant NSCLC cells, by ways of inhibiting MET/Akt/Snail signaling and CAF transformation (Figure 6).

Recently, capmatinib was approved by the FDA for the treatment of patients with metastatic NSCLC harboring a MET exon-14 skipping mutation with disease progression on or after platinum-based chemotherapy. However, a sequential treatment regimen such as the one described in this study has not reported. Further investigation is warranted for the design of future clinical trials.

## MATERIALS AND METHODS

### Cell culture and clinical sample acquisition

Human lung cancer cell lines, H1975 (with activating EGFR L858R+T790M mutations) and HCC827 (E746 - A750 deletion), normal human lung fibroblasts (HLF, PCS-201-013) were obtained from the ATCC (American Type Culture Collection, USA) and maintained according to the conditions suggested by ATCC. Clinical tissues (cancer cells and cancer-associated fibroblasts, CAFs) from patients with non-small cell lung cancer (NSCLC) who have shown resistant and sensitive to osimertinib treatment were collected from Harbin Medical University according to a previously described [18]. The participants were informed and asked to sign consents prior to sample collection. Samples resistant to osimertinib (OR) and sensitive (OS) were maintained and expanded for further experiments. The study was approved by the Institutional Review Board (IRB) of the Harbin Medical University (Harbin, China), consistent with the recommendations of the declaration of Helsinki for biomedical research (Harbin Medical University, Harbin, China) and followed standard institutional protocol for human research. Moreover, the animal study protocol was approved by the Animal Care and User Committee at Harbin Medical University (Harbin, China) (Affidavit of Approval of Animal Use Protocol # Harbin Medical University).

### Bioinformatics analysis

The mRNA expression of MET was analyzed from two databases of lung adenocarcinoma and lung squamous cell carcinoma (generated by the TCGA Research Network: https://www.cancer.gov/tcga). *P<0.05.

### Cell viability test

The IC_50_ values were obtained from cell viability test using Cell Counter Kit-8 (CCK-8, Kumamoto, Japan). Cells were seeded (6.0×10^3^ cells/well) in 96-well plates and incubated with capmatinib (INCB28060, Catalog No.S2788, SelleckChem, USA) followed by osimertinib (AZD9291, Catalog No. S7297, SelleckChem, USA) at indicated concentrations (0 to 30 μM for 48 h) prior to the CCK-8 assay was performed. The OD readings were measured at 450 nm.

### Co-culture experiments and cytokine determination

H1975 and HCC827 cells were co-cultured using a Boyden chamber system, where cancer cells were seeded in the upper chamber (1×10^6^ cells) and CAFs at the bottom (1×10^5^ cells) for 72h. The resultant H1975 and HCC827 cells were termed H1975+CAF and HCC827+CAF and tested with osimertinib (0-30 μM, 48h). In another experimental setting where HLF were co-cultured with OR and OS cells, the upper chamber were seeded with OR or OS cells (1×10^6^ cells) while HLF (1×10^5^ cells) were seeded in the lower chamber. Cells were co-cultured for 72h before harvested for further analyses. The amount of secreted TGFβ1 into the culture medium by CAFs was determined using an ELISA kit (Catalog# OKCD06012, Aviva Systems Biology Corporation, USA); secreted IL-6 by cancer cells into the culture medium was measured using a human IL-6 ELISA kit (Catalog # OKBB00189, Aviva Systems Biology Corporation, USA).

### Lung tumor sphere assay

The protocol for growing lung tumor spheres under serum-deficient conditions was employed to examine the self-renewal ability of the NSCLC cells under different treatment conditions according to a previously established method [31].

### SDS-PAGE and western blots

Total cellular protein lysates from NSCLC cells were exacted and purified using RIPA buffer (Millipore) with a protease/phosphatase inhibitor cocktail (1:100), PMSF (Beyotime). Protein was quantified by a BCA Protein Assay (Thermo Fisher Scientific, Rockford, USA). Samples were run on a 10% SDS-PAGE and transferred to PVDF membranes (Millipore). The membrane was first blocked using PBST (with 5% non-fat milk) washed and incubated with primary antibodies (4° C, overnight). All antibodies were purchased from Cell Signaling Technology, Inc (CTS) unless otherwise specified. p-MET (1:500, CST), p-EGFR (1:500, CST) Vimentin (1:600, CST), Snail (1:1000, abcam), β-catenin (1:1000, CST), MMP9 (1:1000, CST), E-cadherin (1:1000, CTS) and β-actin (1:10000, 60008-1-Ig) in Supplementary Table 1. The protein-antibody complexes were detected using a chemiluminescence kit (ECL; Thermo Fisher Scientific) and captured using an imaging system (Tanon, 5200, China).

### MET silencing and overexpression

The gain- and loss-of-function of MET in OR cells were performed using commercially available reagents. MET silencing was achieved by infecting OR cells with human Met shRNA lentiviral Particles (Cat # sc-29397-V, Santa Cruz, USA), using the protocol suggested the vendor. Gain-of-function of MET was accomplished by infecting OR cells with lentiviral particles containing plasmids of human MET open-reading-frame (ORF, Cat# RC217003L1V, OriGene, USA), according to the protocols provided by the manufacturer. The expression level of MET in the infected OR cells were assayed using western blots.

### Efficacy evaluation of capmatinib and osimertinib combination using PDX model

Here we used patient-derived xenograft mouse model by injecting (osimertinib resistant) OR cells (with matrigel, 7×10^6^ cells) subcutaneously. This experiment was approved by the Animal Care and User Committee at Harbin Medical University (Harbin, China) (Affidavit of Approval of Animal Use Protocol # Harbin Medical University). NSG mice were anesthetized with xilazine (i.p, 8 mg/kg) and ketamine (120 mg/kg) for the tumor injection. Mice were allowed to recover, and tumor growth was monitored. When the tumor became palpable, mice were then randomly divided into 4 groups (N=5 per group) and received different treatments: vehicle group (PBS P.O, 5 times/week), capmatinib alone (10mg/kg, P.O, 5 times/week), osimertinib alone (5mg/kg, P.O, 5 times/week) and combination of both drugs (combined both regimens). The tumor growth was monitored and measured using a standard caliper. The tumor volume is calculated using the formula: V= (L×W^2^)/2, L is the long axis and W is the width. Mice at the end of the experiment were humanly sacrificed and tumor samples were collected for further analyses.

### Immunostaining of tumor sections

Tumor samples were collected from the mice and fixed by formalin, followed by paraffin-embedment. Microsections were prepared (5-μm thickness) for staining protocol. Sections were dewaxed using xylene and rehydrated with serial ethanol gradients and finally rinsed in deionized water. Sections were blocked in PBS with 1% donkey serum and 0.3% Triton X-100 and 0.01% sodium-azide for 1h, room temperature. Primary antibodies prepared in diluted in blocking buffer were applied the sections and incubated in cold overnight. Next day, sections were washed in PBST 3 times followed by incubation with secondary antibodies in blocking buffer (1h, room temperature). Sections were then stained using DAB chromagen (ThermoFisher Scientific, USA) and mounted. The slides were observed under a microscope and micrographed using an inverted TE 2000 wide-field microscope system (Nikon, Japan).

### Statistical analysis

All experimental data was expressed as mean + SD (standard deviation) and graphed using GraphPad Prism software (GraphPad Software, Inc., CA, USA). T test was used to show the difference between two experimental groups. P<0.05 is recognized as statistically significant. All experiments were repeated at least three times.

### Availability of data and materials

The datasets used and analyzed in the current study are available from the corresponding author in response to reasonable requests.

### Ethics approval and consent to participate

Clinical samples were collected from Harbin Medical University (Harbin, China). All enrolled patients gave written informed consent for their tissues to be used for scientific research. The study was approved by the Institutional Review Board (IRB) of the Harbin Medical University (Harbin, China), consistent with the recommendations of the declaration of Helsinki for biomedical research (Harbin Medical University, Harbin, China) and followed standard institutional protocol for human research. Moreover, the animal study protocol was approved by the Animal Care and User Committee at Harbin Medical University (Harbin, China) (Affidavit of Approval of Animal Use Protocol # Harbin Medical University-LAC-0001).

## Supplementary Material

Supplementary Table 1
