# Supplementary material for: MET inhibitor, capmatinib overcomes osimertinib resistance via suppression of MET/Akt/snail signaling in non-small cell lung cancer and decreased generation of cancer-associated fibroblasts
Source: Aging (Albany NY). 2021 Feb 17;13(5):6890–903. doi: 10.18632/aging.202547 (PMC7993678; doi:10.18632/aging.202547)
Supplement: Supplementary Table 1 [file aging-13-202547-s001.pdf]

SUPPLEMENTARY TABLE

Supplementary Table 1. Western blot antibodies sheet.

No.	Target	Dilution	Source	
1	Vimentin	1:1000	#5741	Cell signaling
2	β-catenin	1:1000	#8480	Cell signaling
3	MMP9	1:1000	#13667	Cell signaling
4	E-cadherin	1:1000	#14472	Cell signaling
5	p-MET	1:500	#3077	Cell signaling
6	MET	1:1000	#8198	Cell signaling
7	p-EGFR	1:500	ab40815	Cell signaling
8	p-Akt	1:500	ab38449	abcam
9	Snail	1:1000	ab216347	abcam
10	α-SMA	1:1000	ab7817	abcam
11	β-actin	1:10000	10494-1-AP	60008-1-Ig
